# Percutaneous Trapeziometacarpal Arthrodesis in Thumb Carpometacarpal Joint Arthritis: A New Surgical Approach

**DOI:** 10.1155/2021/6881168

**Published:** 2021-10-27

**Authors:** D. Mifsut-Miedes, J. R. Rodríguez-Collell, A. Valverde-Navarro, E. M. González-Soler

**Affiliations:** ^1^Universitat de València, Spain; ^2^Hospital Malvarrosa de Valencia, Spain

## Abstract

The high prevalence of trapeziometacarpal arthritis has resulted in the development of several surgical techniques intended to treat patients failing conservative treatment. However, there is no scientific evidence of the superiority of one technique over others. Open arthrodesis has up to now been successfully used to treat this condition. We believe that performing the technique using a minimally invasive approach with long and short Shannon burrs together with the tapered burr included in the MIS foot instrument set can yield satisfactory results. This article provides a description of this minimally invasive technique performed on a seventy-year-old woman with rhizarthrosis and an anatomical description of the approach in a human cadaver.

## 1. Introduction

Osteoarthritis of the trapeziometacarpal joint of the thumb is a common condition affecting 1 in every 4 women and 1 in every 12 men [1]. In each case, surgeons select the operative technique to be used as a function of the patient's age, comorbidities, functional demand, and radiographic stage of their condition. Options include ligament reconstruction, metacarpal osteotomy, arthroscopy and debridement, total joint replacement, silicone arthroplasty, trapeziometacarpal arthrodesis (TMA), and excision of the trapezium with or without ligament reconstruction and tendon interposition (LRTI).

Multiple systematic reviews have demonstrated that no surgical technique is superior to the others [2, 3]. Arthrodesis may be preferable in younger and more active patients with moderate to severe arthritis of the trapeziometacarpal joint of the thumb who are required to use force and grasp objects as part of their job, even for older patients. Complications associated with arthrodesis include nonunion (7-16%), hardware-related complications, and a proneness to arthritis in the adjacent joints, with an associated loss of range of motion and an inability to hold the hand flat. On the other hand, trapeziectomy could potentially result in shortening of the first ray, which may in turn decrease thumb force, or painful subluxation or arthritis of the pseudoarthrosis between the metacarpal base and the scaphoid [1]. The arthroscopic approach is an alternative in the treatment of trapeziometacarpal arthritis but above all to perform arthroscopic synovectomy/debridement/thermal shrinkage, arthroscopic partial trapeziectomy, and suture button suspensionplasty, etc. Despite such complications, the subjective and objective outcomes of arthrodesis are similar to those of arthroplasty.

The aim of this paper is to describe a new percutaneous TMA technique where the arthrodesis is fixed by means of two self-tapping cannulated screws, in a patient diagnosed with rhizarthrosis, with a good final result. We show an anatomic dissection in a human cadaver with this approach.

## 2. Clinical Case

A seventy-year-old woman was admitted to the orthopaedic outpatient department complaining of pain and swelling at the first ray of the right hand that had been occurring over the preceding two years. Furthermore, she described feeling weak when trying to catch or hold objects, including serious difficulties in carrying out basic daily activities. There was no previous history of trauma. The patient had worked as a hairdresser and was right-handed, with a personal history of hypertension under treatment.

On physical examination, pain and crepitation were noted in the metacarpophalangeal joint of the thumb, which appeared swollen with a positive “shoulder deformity.” In addition, the pain worsened with compression on the axis of the first metacarpal together with a simultaneous circumduction movement (Crank test) and a rotational movement (Grind test). A compensatory deformity, such as a 25° hyperextension of the metacarpophalangeal joint of the thumb, was found. This abnormality did not decrease with passive abduction of the trapeziometacarpal joint. As for mobility, major limitation was observed in the opposition of the thumb. The patient displayed no signs of carpal tunnel syndrome or other entities such as trigger fingers or De Quervain's tenosynovitis.

The mean value of the Disabilities of the Arm, Shoulder and Hand Questionnaire (DASH score) corresponded to severe difficulty in performing daily activities or severe symptomatology (66). The value of the visual analog scale was 8 out of 10.

Radiographic assessment demonstrated a marked impingement of the trapeziometacarpal joint with a total loss of contour of the articular surfaces and the presence of subchondral cysts with a subluxation of the joint surface greater than a third. Osteophyte size was greater than 2 mm.

**Figure 1:**
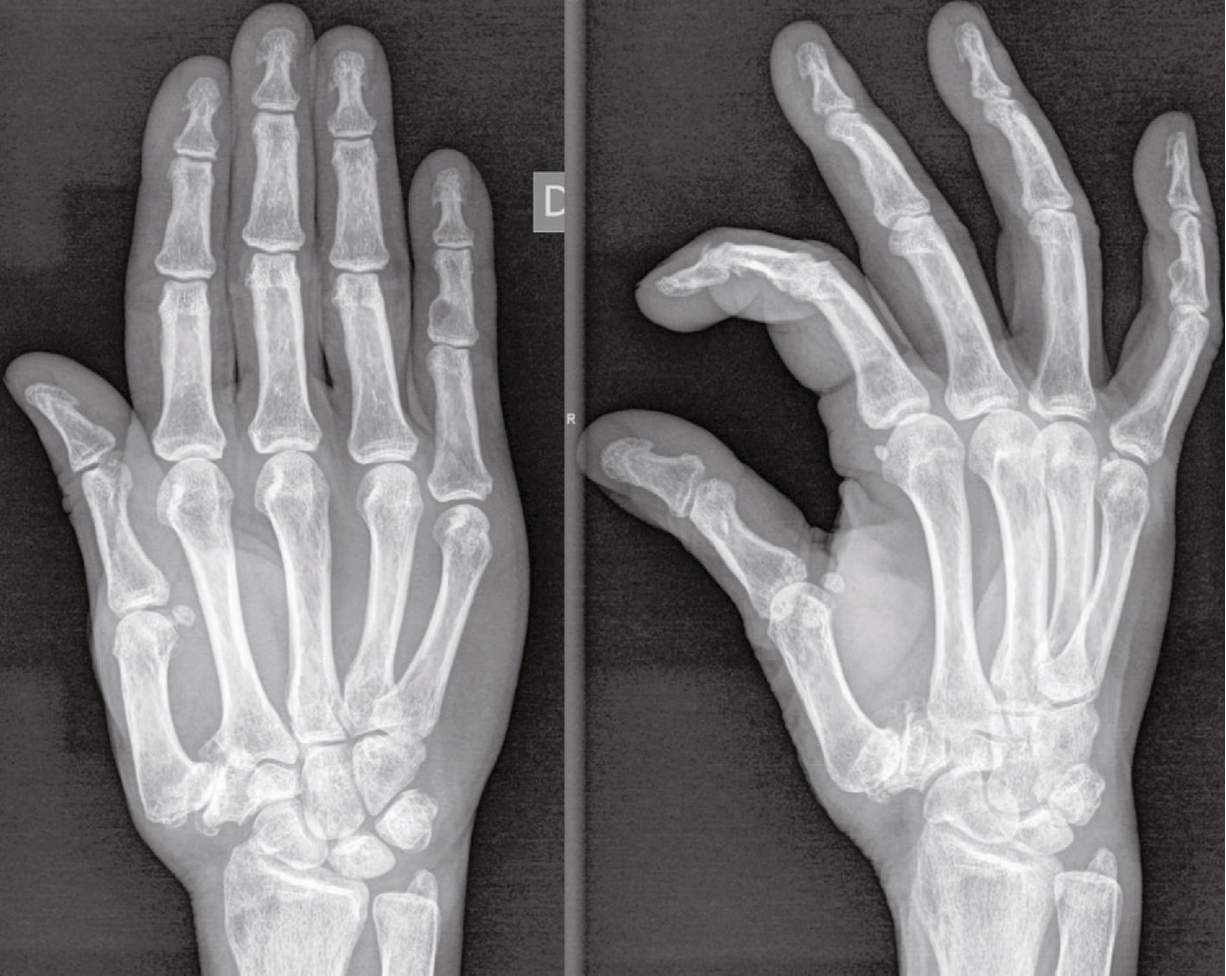
Initial X-ray showing trapeziometacarpal osteoarthritis.

Thus, this was a case of rhizarthrosis Grade IV according to the classification of Eaton, associated with hyperextension of the metacarpophalangeal joint (Figure 1). As a result of the dysplastic morphology of the trapezium, conventional arthroplasty with trapeziometacarpal prosthesis was ruled out. Surgery for advanced rhizarthrosis was scheduled, and a percutaneous arthrodesis was performed as described below.

## 3. Surgical Technique

**Figure 2:**
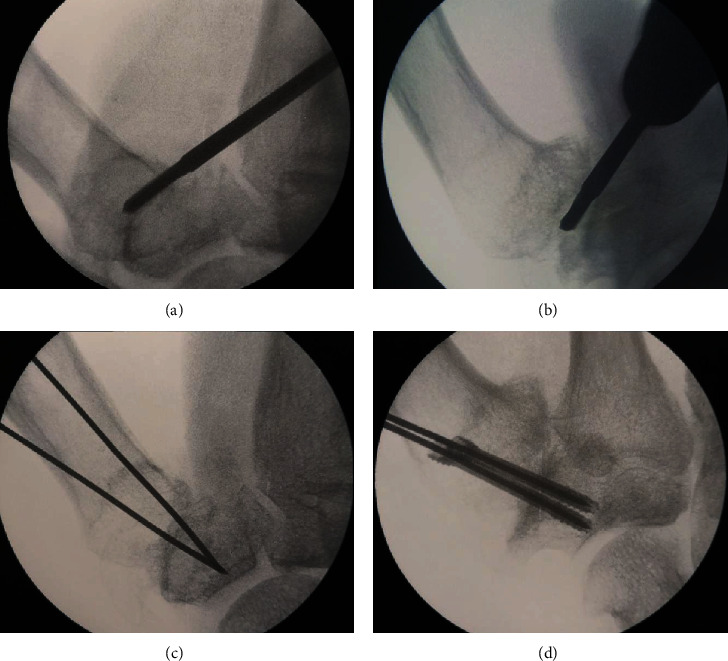
Minimally invasive approach to the ulnar aspect of the trapeziometacarpal joint (a, b). Anatomical dissection shows the entry point and Shannon burr access (c, d).

The operation was carried out under axillary brachial plexus block anaesthesia. A MS64 scalpel was used to perform a minimally invasive approach to the ulnar aspect of the trapeziometacarpal joint, laterally to the extensor pollicis longus. Subsequently, the short and long Shannon burrs and the tapered burr were introduced under fluoroscopic control (Figure 2) in order to debride the joint surface. From this position, access to the medial osteophyte is relatively straightforward (Figures 2(a) and 2(b)).

After the first stage of the procedure, two thin K-wires were inserted as guidance to fix the trapeziometacarpal joint (Figure 2(c)) with two SpeedTip CCS 3.0 cannulated compression screws (Medartis®) from the dorsal aspect of the metacarpal joint to the trapezium, also under fluoroscopic control (Figure 2(d)). After this, the K-wires were removed, and the wound was closed.

**Figure 3:**
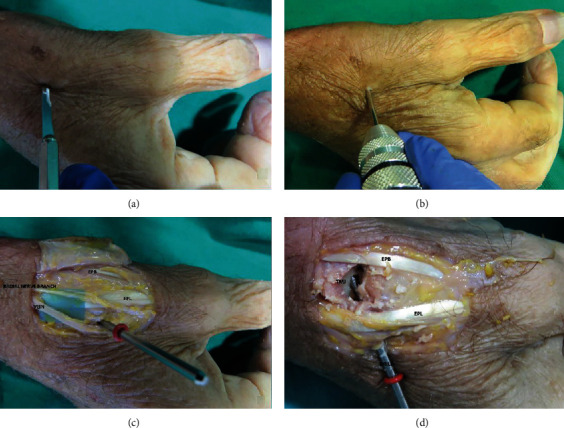
Anatomical dissection in human cadaver: short Shannon burr introduction for surface curettage (a, b). Two thin K-wires were inserted as guidance to fix the trapeziometacarpal joint (c), and cannulated compression screws were inserted (d).

The anatomic dissection shows the opening through which the burrs were introduced. As the entry point was close to the sensitive branch of the radial nerve and the dorsal venous network, a gentle blunt dissection was performed in that area (Figures 3(a)–3(c)). With this approach, we have good access to the joint (Figure 3(d)).

## 4. Results

A volar splint including the first ray was placed for a period of 3 weeks. After this time, the patient was referred to the rehabilitation service, and three months after surgery, she returned to her usual activity and was free of pain.

**Figure 4:**
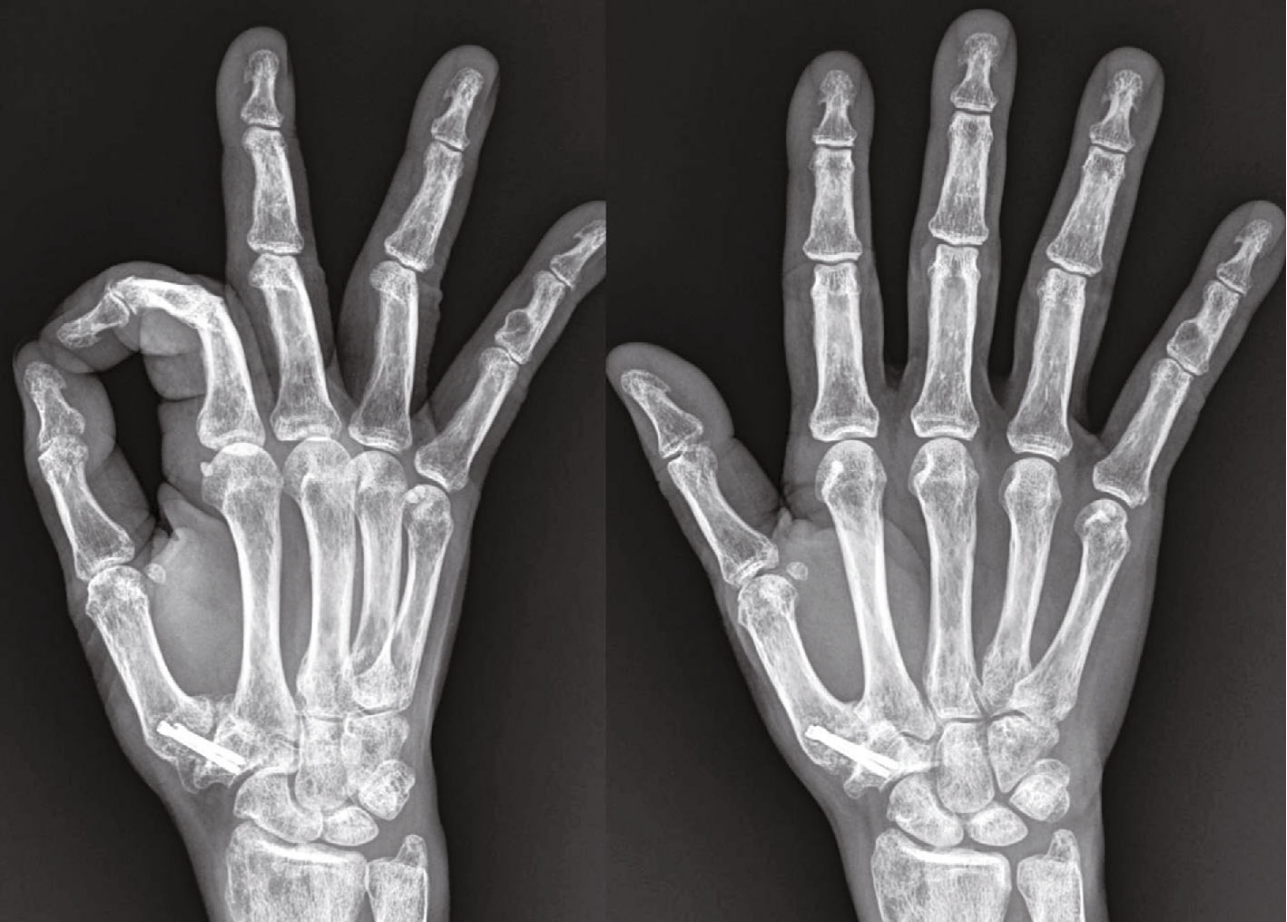
Final radiograph showing the arthrodesis of the trapeziometacarpal joint of the right hand.

The patient remained without symptomatology, and the metacarpophalangeal joint continued to be stable, with an opposition movement up to the distal volar crease and an opening of the first commeasure of more than 50°. The mean value of the DASH score at follow-up was between the absence of symptoms and the slightest degree of discomfort (12, 5). The final X-ray control, after two years, showed total arthrodesis trapeziometacarpal (Figure 4).

## 5. Discussion

There is widespread consensus that treatment of arthritis of the trapeziometacarpal joint of the thumb is chiefly aimed at relieving pain, increasing force, and providing the thumb with an appropriate range of motion and sufficient stability. However, the best treatment to achieve such goals is still a matter of debate.

Multiple systematic reviews have been conducted without any of them succeeding in gathering enough scientific evidence supporting one technique over the others [2, 3]. Arthrodesis of the trapeziometacarpal joint allows the thumb to perform a strong pinch with greater stability than other procedures, being considered one of the techniques of choice for treating young patients requiring increased thumb force to do their job. Some recent series recommend its use even in patients over 40 years of age. Proubasta [4] described an extra-articular arthrodesis technique which did not require debridement of the joint surfaces and where the graft is fixed by means of two Herbert screws. Duerinckx and Wolff [5] described a novel technique to shape the joint surfaces, which provided inherent stability and a larger contact area, making it easier to place the thumb in an ideal position for the arthrodesis.

The results of trapeziometacarpal arthrodesis have greatly depended on the fixation technique employed. Having said this, we believe that—as with other minimally invasive procedures—percutaneous arthrodesis constitutes a better alternative than its open counterpart as it results in decreased soft tissue damage, lower nonunion rates (as the debrided tissue is retained at the site of arthrodesis), and faster postoperative patient recovery.
